# The recent progression of extracellular vesicles application in osteoporosis 

**DOI:** 10.3389/fphar.2026.1843835

**Published:** 2026-06-16

**Authors:** Rui Liu, Wenyu Li, Junzheng Yang

**Affiliations:** 1 School of Life Sciences and Biotechnology, North Henan Medical University, Xinxiang, Henan, China; 2 Guangdong Nephrotic Drug Engineering and Technology Research Center, Guangzhou, China

**Keywords:** application, extracellular vesicles, osteoporosis, pathogenesis, risk factors, underlying mechanism

## Abstract

Osteoporosis is a type of chronic disease that leads to elevated bone fragility and increased risk of fractures. The principal characteristics of this condition include reduced bone density and bone quality, impaired bone microstructure, and increased vulnerability to fractures. These fractures frequently occur in elderly individuals, resulting in significant health impairment and disruption to their daily lives. The economic consequences of osteoporosis and osteoporosis-related fractures can be substantial. Extracellular vesicles (EVs) are a category of small particles that are secreted by cells under both normal and pathological conditions. These particles contain various active substances, including proteins, lipids, and nucleic acids. These elements play a pivotal role in various processes within the human body, including cell growth, differentiation, and apoptosis. Recent studies have indicated that EVs have also made significant progress in the field of osteoporosis, demonstrating considerable potential for application. In this review, we summarize the pathogenesis and risk factors of osteoporosis, and the therapeutic and diagnostic application of EVs in osteoporosis and the underlying mechanisms. Furthermore, the current limitations of applications and potential solutions are also discussed. This review will provide researchers with new insights and research direction in the future.

## 1 The epidemiology of osteoporosis

**FIGURE 1:**
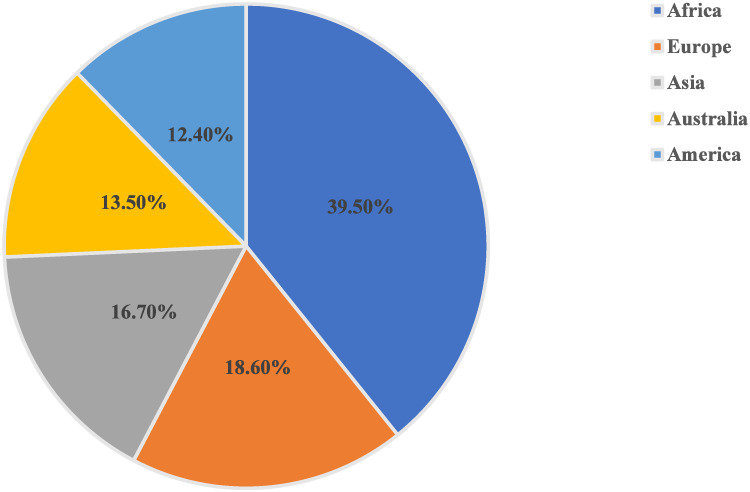
The summary of the global epidemiology of osteoporosis (Salari et al., 2021).

Osteoporosis is defined as a chronic systemic orthopedic disease. The typical clinical manifestations of osteoporosis include pain, spinal deformation and brittle fracture (Yao et al., 2025). It has been demonstrated that osteoporosis predominantly afflicts middle-aged and elderly individuals, with its incidence rate exhibiting an age-related increase (Girdler et al., 2024; Ye et al., 2024). The absence of overt symptoms in the early stages of the disease complicates diagnosis, leading to a high prevalence of undiagnosed osteoporosis and a low rate of treatment (Thomasius et al., 2025). A comprehensive meta-analysis demonstrated that the global prevalence of osteoporosis is 18.3%, and the incidence rate of osteoporosis in women is significantly higher than that in men (23.1% vs. 11.7%). On a global scale, Africa exhibits the highest incidence rate, followed by Europe, Asia, and Oceania, while North America demonstrates the lowest incidence. The influencing factors include environment, lifestyle habits, and race (Salari et al., 2021) (Figure 1). A study of epidemiological data has revealed that the number of individuals diagnosed with osteoporosis in China is approximately 90 million, of whom 78% are female. The prevalence of osteoporosis in the population aged 50 and above is approximately 20%, with a higher incidence observed in females (32%) compared to males (7%). A more pronounced increase in the prevalence of osteoporosis is observed among the female (52%) and male (11%) population aged 65 and above, indicating disparities with regard to age and gender (Fan et al., 2024; Yu and Xia, 2019). A further statistical analysis has estimated that the number of osteoporosis-related fractures is anticipated to reach 5 million by 2035, with the resultant medical expenses amounting to approximately 20 billion dollars per annum. This has a significant impact on the quality of life of patients with osteoporosis and also exerts a considerable economic pressure on them (Chen et al., 2022; Dong et al., 2022).

Furthermore, research has demonstrated a correlation between osteoporosis and the occurrence of fractures, which can have a significantly detrimental effect on health (Nakatoh et al., 2026). Furthermore, it has been demonstrated that fractures are associated with elevated rates of disability and mortality among elderly patients, frequently precipitating a range of complications and engendering a substantial deterioration in their quality of life (Li L. et al., 2025). The medical expenses it generates will also impose a heavy burden on families and society (Sund et al., 2025). According to statistical data, Statistical data indicates that by 2035, the expenditure on medical treatment for major osteoporotic fractures in China is projected to exceed 130 billion. By the year 2050, it is predicted that this segment of medical expenditure will rise by almost 170 billion (Chi et al., 2026). The findings of this study indicated that osteoporosis has a significant population prevalence in China and worldwide, which imposes a substantial economic burden on the prevention and treatment of this condition (Girgin et al., 2025).

## 2 The functions of EVs

Extracellular vesicles (EVs) are a type of lipid membrane-like structures with a diameter range of ranging from 10 to 5000 nm. They are capable of transporting various functional active molecules, including proteins, nucleic acids, and metabolites, thereby facilitating signal transmission between cells (Anwar et al., 2026; Shi et al., 2025; Forero et al., 2024). It has been demonstrated that EVs possess bidirectional communication capabilities, the capacity to absorb environmental substances and the ability to secrete active molecules. In detail, EVs are capable of transporting a variety of substances, including miRNAs, lipids and proteins. Receptor cells then take up the outer vesicles through the process of endocytosis. The outer vesicles subsequently fuse with the cell’s internal or external membranes, releasing their contents into the cytoplasm where they can exert their functions (Hrdinova and Kupcova Skalnikova, 2026). Research has demonstrated that EVs play a role in the occurrence, development, and treatment of osteoporosis by mediating the “dialogue” between the bone, the immune, and muscle systems (Yang L. et al., 2025). For example, the EVs secreted by osteoblasts carry miR-150-5p, which can effectively inhibit the formation of osteoclasts and prevent excessive bone loss. The outer vesicles secreted by macrophages contain pro-inflammatory factors, which can create an inflammatory environment, promote osteoclastogenesis, and accelerate bone destruction (Carney et al., 2025; Hu S. J. et al., 2025; Zhang et al., 2024).

EVs are lipid bilayer structures that are derived from cells and have the ability to encapsulate nucleic acid substances, thereby reducing their degradation and improving their stability (Shinde et al., 2024). The presence of numerous immune regulatory factors on the outer vesicles serves to render them less immunogenic and to reduce immune rejection reactions (Shimizu et al., 2025). The secretion of EVs is a process that is almost universally observed in cells, thus facilitating the collection of large quantities of EVs (Gilboa et al., 2025). EVs are characterized by their small size, biocompatibility, inherent targeting ability, low immunogenicity, and capacity to safeguard cargo from enzymatic degradation. Consequently, EVs possess considerable application potential in a wide range of diseases, including osteoporosis (Anand et al., 2025; Schweer et al., 2023; Sun et al., 2024; Liu et al., 2024b). For example, Mizukami et al. (2026) demonstrated that MC3T3-E1 cell-derived EVs improved the bone mineral density and bone volume in osteoporosis mice, and identified EVs encapsulated miR-1224-5p as the key therapeutic components for osteoporosis (Mizukami et al., 2026). Shao et al. (2026) have identified miR-125a-5p as an important component in the process of the treatment of EVs derived from atrophic skeletal muscle in osteoporosis mice. The data demonstrated that the overexpression of miR-125a-5p in skeletal muscle will exacerbate the symptoms of osteoporosis, including muscle atrophy and bone loss, and inhibited the expression of miR-125a-5p could reverse these side effects (Shao et al., 2026). In this review, we will summarize the pathogenesis and risk factors of osteoporosis. We then proceed to discuss the preclinical applications of EVs in osteoporosis and the underlying mechanisms. We also consider the current application limitations and potential solutions. It is our hope that our review will provide researchers with new insights in the future.

## 3 The pathogenesis and risk factors of osteoporosis

Osteoporosis is a type of chronic disease characterized by decreased bone mass and loss of bone tissue microstructure (Laurent et al., 2025). The human skeleton is subject to a dynamic process of long-term changes. Before reaching adulthood, the human body’s bones undergo a continuous process of development and maturation. During this process, the formation of new bone tissue exceeds the resorption of existing bone tissue, thereby resulting in an overall increase in the body’s bone mass. Following the attainment of adulthood, the human body’s bones reach complete maturity, and the balance between bone formation and absorption becomes dynamic (Xie et al., 2025; Qian et al., 2025). The fundamental pathogenesis of osteoporosis is characterized by an imbalance between the processes of bone resorption and bone formation during bone metabolism (Bartosik et al., 2026). This imbalance can be attributed to the over-activation of osteoclasts, resulting in the excessive removal of bone tissue, or the under-activation of osteoblasts, leading to an insufficient repair of the bone cavities (Terhaar et al., 2025). The core concern regarding osteoporosis pertains to osteoporosis is the disruption of the dynamic balance between the processes of “bone formation” by osteoblasts and the “bone resorption” by osteoclasts (Chen et al., 2026). The Wnt/β-catenin pathway is the core switch that regulates osteogenic differentiation. Under normal circumstances, the β-catenin protein accumulates within cells and enters the nucleus, initiating the expression of osteogenic related genes (such as Runx2 and OCN). The inhibition of the Wnt/β-catenin signaling pathway can lead to the loss of osteoblast function (Pramoda et al., 2026). It has been demonstrated that DKK1 can inhibit the expression of Wnt, while USP26’s deubiquitinase can protect β-catenin from degradation (Lin et al., 2025). Specifically, the skeletal protein of MACF1 can retain negative regulatory factors for osteogenesis in the cytoplasm, while BMP9 promotes osteogenic differentiation through the mTORC1/STAT3 signaling pathway (Su et al., 2025; Zhang H. et al., 2025). Osteoclasts are derived from monocytes macrophages in the blood. RANKL is considered a key factor in activating osteoclasts, as it activates signaling pathways such as NF-κB, leading to the production of a large number of mature osteoclasts (Zhu M. et al., 2026). Research has identified a role for USP26 in the inhibition of osteoclast differentiation. In cases where USP26 is not sufficient, IκBα is degraded and the NF-κB pathway is released. This leads to the production of a large number of osteoclasts and result in osteoporosis (Fan et al., 2024). Further evidence suggests that mitochondrial energy metabolism and mitochondrial autophagy can also promote osteoclast differentiation, exacerbate inflammatory responses, and promote excessive formation of osteoclasts (Zhang J. et al., 2026).

It is well established that multiple hormones are involved in the pathogenesis of osteoporosis (Tang H. et al., 2025). These hormones are vital for maintaining a dynamic balance between regulating bone formation and resorption, and regulating the progression of osteoporosis (Borges et al., 2026). Research has demonstrated a correlation between sex hormones and bone strength. The role of estrogen in regulating bone metabolism is characterized by a typical unidirectional protective effect. A decrease in sex hormone levels, however, can result in primary osteoporosis (Sheng et al., 2023). Research has been demonstrated that estrogen levels can influence the formation of osteoclasts by promoting the secretion of osteoprotegerin (OPG); it can also regulate the Wnt signaling pathway to impact the survival and function of osteoblasts (Lee et al., 2026). Further evidence demonstrated that estrogen plays a role in protecting osteoblasts from oxidative stress damage by maintaining the osteoclasts and osteoblasts coupling (Wang S. et al., 2026). Androgens regulate bone metabolism in two main ways. (1) Androgens bind with androgen receptors (AR) on the surface of bone cells and osteoblasts, regulating the functions of these cells (Michalopoulou et al., 2026). (2) Androgens undergo aromatization into estrogens, which regulate bone metabolism through estrogen receptors (Junnila et al., 2025). Calcium-regulating hormones, including parathyroid hormone (PTH), vitamin D, and calcitonin, are the main factors in maintaining calcium-phosphorus balance in the body and regulating bone mineralization and intestinal calcium absorption (Zhang et al., 2021; Yadav et al., 2026). Specifically, PTH has been found to have a bidirectional regulatory effect on bone metabolism (Alhumaidan and Elakel, 2026). When hyperparathyroidism or severe vitamin D deficiency occurs, there is an increase in PTH levels, resulting in elevated RANKL protein production and reduced OPG protein expression in bone cells. This results in the abnormal activation of a large number of osteoclasts, causing a rapid loss of bone mass (Dai et al., 2026). Intermittent PTH results in a brief increase in PTH in the blood, followed by a rapid decrease. This pulsed stimulation has been shown to cause a brief increase in RANKL, preferentially activating osteoblasts, resulting in bone formation exceeding bone resorption, thereby achieving therapeutic net bone mass growth (Coman et al., 2025). On the one hand, PTH exerts a direct effect on osteoblasts, acting via PTH receptors present on their surface. This leads to an enhancement of osteoblast differentiation and survival (Modiz et al., 2025). On the other hand, PTH has been shown to promote the production of osteocalcin, which in turn activates the Wnt signaling pathway and increases bone volume. This results in a further increase in new bone formation (Zhang W. et al., 2025).

In recent years, research has revealed that pituitary hormones can also act on osteoblasts or osteoclasts, thereby regulating bone metabolism (Chen M. et al., 2025). It is widely accepted that pituitary hormones influence bone metabolism by regulating the secretion of hormones from target glands, including thyroid and gonads (Wang G. et al., 2026). Recent studies have found that pituitary hormones can directly act on bone cells and exert their effects (Chen X. et al., 2025). Furthermore, studies have found that immune cells in the bone marrow can also produce variants of pituitary hormones (such as TSH-βv) that act on bone cells and participate in bone metabolism (Ki et al., 2024).

Gut microbiota is a complex microbial ecosystem within the human body that regulates the occurrence and development of osteoporosis through the gut-bone axis (Huang Y. et al., 2026). Research has demonstrated that gut microbiota can influence the balance of Th17/Treg cells. In the absence of underlying pathologies, the gut microbiota is responsible for maintaining cell activity, thereby promoting bone health (Wu et al., 2026). However, an imbalance in the gut microbiota can trigger an inflammatory response, resulting in increased bone resorption relative to bone formation (Zhang Y. et al., 2026). Furthermore, it has been demonstrated that metabolites produced by gut microbiota also play an important role in the development of osteoporosis. Research has indicated that short-chain fatty acids present within the gut microbiota can inhibit the differentiation and activity of osteoclasts, exerting a protective effect on bones (Zhu S. et al., 2026). In addition, secondary bile acids can inhibit the expression of NF-κB protein, which in turn suppresses osteoclast maturation (Hassan et al., 2026). Furthermore, research has demonstrated that gut microbiota can participate in regulating estrogen metabolism and increasing the level of active vitamin D in the blood, which is involved in the occurrence of osteoporosis (Wu et al., 2026).

Interleukin-17(IL-17) has been identified as a pivotal inflammatory factor in the connection between the immune system and bone metabolism (Chen Y. et al., 2025). Evidence has demonstrated that IL-17 promotes the generation of “osteoclasts” in osteoporosis through the immune-skeleton regulatory network (Li D. et al., 2026). RANKL is the main regulatory factor for the formation and function of osteoclasts. Research has found that IL-17 can enhance the sensitivity of precursor cells of osteoclasts to RANKL signaling, thereby promoting their differentiation into mature osteoclasts (Zhou et al., 2026). Furthermore, IL-17 can upregulate the expression of RANKL and to activate the RANKL/RANK signaling pathway, affecting the occurrence and development of osteoporosis (Rao and Cai, 2026). In addition, IL-17 can activate various inflammatory signaling pathways, including NF-κB and JAK/STAT3, which collectively promote bone destruction and exacerbate bone loss (Lu J.S. et al., 2025; Pi et al., 2025).

**FIGURE 2:**
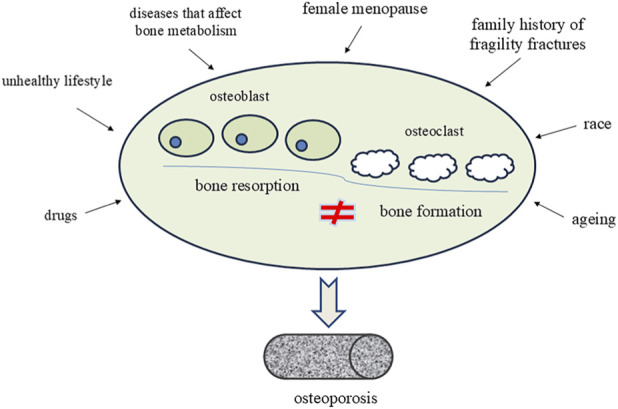
The summary of risk factors of osteoporosis.

Osteoporosis is a multifaceted disease influenced by a multitude of genetic and environmental factors. Several types of risk factors have been identified as contributors to the loss of bone mass in the human body, ultimately leading to the development and progression of osteoporosis. These factors encompass both controllable and uncontrollable factors. The former includes unhealthy lifestyle choices, diseases that affect bone metabolism, and drugs that have a similar effect. The latter include factors such as race, ageing, female menopause, and family history of fragility fractures (Figure 2). For example, it has been demonstrated that the lack of sex hormones is capable of impeding the osteoclast activity, resulting in the degradation of bone cells and the subsequent loss of bone tissue. This, in turn, can have a detrimental effect on the development and progression of osteoporosis (Hu Y. et al., 2025). Similarly, the process of ageing has be identified to be associated with a decline in sex hormones, an insufficiency of vitamin D, a chronic negative calcium balance, the inhibition of osteoblasts, and a reduction in bone mass, which can also contribute to the progression of osteoporosis (Hudson et al., 2024).

**FIGURE 3:**
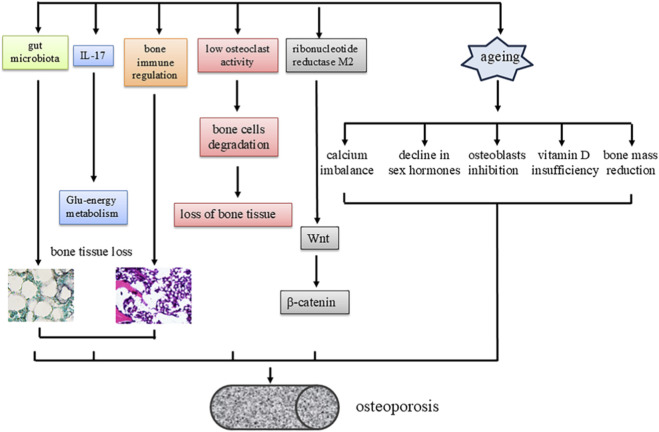
The summary of the pathogenesis of osteoporosis.

Furthermore, abnormal regulation of bone metabolism, or calcium metabolism, in conjunction with a number of types of metabolic disorder, and the administration of certain medications, have the capacity to disrupt the balance between bone formation and resorption, thereby exerting an effect on the process of osteoporosis (Li Y et al., 2025; Paccou et al., 2024). Recent studies have indicated the pivotal function of gut microbiota in the development of osteoporosis (Hao et al., 2025). Qin, et al. have demonstrated the involvement of bone immune regulation in the pathogenesis of osteoporosis through the regulation of NF-κB pathway (Qin et al., 2024). Furthermore, Cai, et al. have shown that ribonucleotide reductase M2 (RRM2) promoted the osteogenic differentiation in mouse embryo fibroblasts by regulating the Wnt/β-catenin signaling pathway, thus providing a potential therapeutic method for osteoporosis (Cai et al., 2022). Peng et al. (2024) found that glutamine plays a role in the differentiation and function of osteoclasts. Furthermore, glutamine has been found to participate in the process of IL-17 in promoting osteoclast differentiation and in regulating energy metabolism. These findings suggest that IL-17-Glu-energy metabolism may be a potential therapeutic target for the treatment of osteoporosis (Peng et al., 2024) (Figure 3).

## 4 The biogenesis and classification of EVs

EVs are a type of membrane-bound vesicles that are released from cells into the extracellular environment. They play important roles in intercellular communication, immune regulation, and disease transmission (Harvey et al., 2022; Chen M. et al., 2024; Dlugolecka et al., 2025; Weiss et al., 2025; Rutter et al., 2026). Extensive research has revealed the presence of EVs in various biological fluids, including blood, urine, saliva, and cerebrospinal fluid (You et al., 2023; Malhotra et al., 2023). In accordance with the International Society of EVs (ISEV)’s MISEV Guidelines, EVs can be classified into various types, including exosomes, microvesicles, apoptotic bodies (Zhou et al., 2023). It is important to note that exosomes are a type of membrane-bound vesicle with a size of 40–150 nm. These particles originate from the inner membrane system and play an important role in intercellular communication (Kimiz-Gebologlu and Oncel, 2022). Microvesicles are a subtype of EVs with a size of 100 nm-10 µm. These particles mainly form through direct budding from the cytoplasmic membrane and are involved in various pathophysiological functions (Mobarak et al., 2024). Apoptotic bodies are produced by membrane foaming during programmed cell death. These bodies have a diameter ranging from 50 nm to 5 µm and contain DNA and organelle components. It has been established that these bodies participate in various physiological functions, including cell apoptosis (Yu et al., 2023).

**FIGURE 4:**
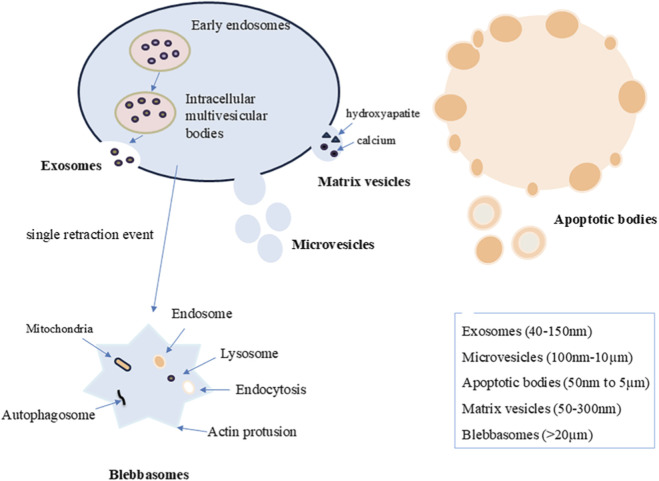
The biogenesis and classification of EVs.

In accordance with the most recent and authoritative International Society of EVs (ISEV)’s MISEV Guidelines, the classification of EVs cannot be exclusively based on “particle size” or “source” as the sole criteria for classification. A more accurate approach would be to describe EVs based on their “physical properties” and “biochemical characteristics”. Need to mentioned that matrix vesicles are a type of EVs that have been identified that concentrate calcium and inorganic phosphate, and had the effect of transporting calcium and phosphate to the collagen matrix. Especially, they are primary secreted by osteoblasts and chondrocytes which encapsulated several key components for bone mineralization, including phospholipids and alkaline phosphatase (TNAP), exhibit the potential effect on osteoporosis (Clews et al., 2025). Recently, a type of EVs with a diameter of up to 20 µm has been discovered and named blebbasome. This type of EVs has bidirectional vesicle interaction function, can uptake extracellular vesicles, secrete exosome and microvesicles, and play a role in various physiological processes (Jeppesen et al., 2025) (Figure 4).

## 5 The therapeutic effect of EVs in osteoporosis

Due to the unique application advantages of EVs, many studies have demonstrated the positive therapeutic effects of EVs in the treatment of osteoporosis. For example, Ma et al. (2023) isolated the skeletal muscle-derived EVs using a total exosome isolation reagent kit. They discovered that these EVs have the capability to promote the osteogenic differentiation of BMSCs and to stimulate the bone formation in osteoporosis mice, indicated that the skeletal muscle-derived EVs may be a potential therapeutic method for treating osteoporosis (Ma et al., 2023). Wang et al. (2024) compared the effect of young osteocyte-derived extracellular vesicles (YO-EVs) and senescent osteocyte-derived EVs on senile osteoporosis in mice. The results demonstrated that young osteocyte-derived extracellular vesicles exhibited the higher physiological activity, including improving the bone mass and biomechanical strength, by regulating tropomyosin-1 (TPM1) (Wang et al., 2024).

**FIGURE 5:**
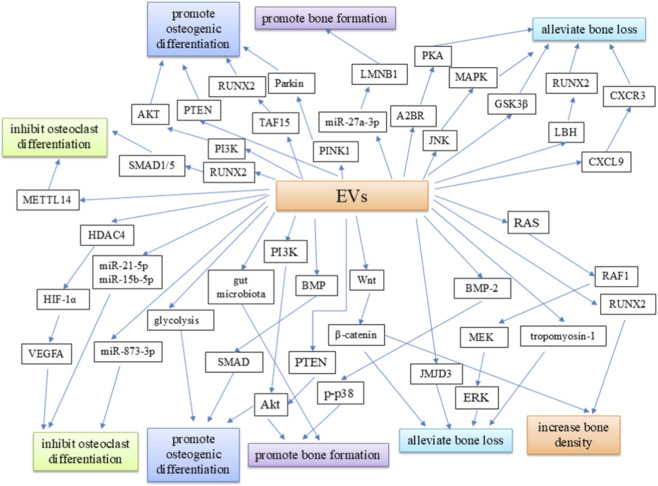
The summary of the mechanism of therapeutic effect of EVs in osteoporosis.

In order to further enhance the therapeutic effect of EVs, Kang et al. (2024) developed a type of exosomes which were incorporated with phosphatidylserine (PS). These exosomes exhibited the characteristic of affinity to osteoclast precursors and anti-resorptive effects, and demonstrated a superior effect in decreasing bone loss in osteoporosis mice. The therapeutic process was regulated by inhibiting the RANKL-induced NF-κB signaling and CXCL9-CXCR3 axis (Kang et al., 2024). Similarly, EVs derived from plants demonstrated therapeutic benefits in the treatment of osteoporosis. Cao et al. (2024) obtained the Morinda Officinalis-derived extracellular vesicles (MOEVs) by differential centrifugation and ultracentrifugation method, and evaluated the effect of MOEVs in the postmenopausal osteoporosis (PMOP) mouse model. The data demonstrated that MOEVs could regulate the expressions of the expression of RUNX2 and OCN and improve the bone formation in mouse. Furthermore, MOEVs also exhibited promotion effect on the proliferation of MC3T3-E1 cells by regulating MAPK pathway (Cao et al., 2024). Zhan et al. (2023) assessed the effect of exosome-like nanovesicles derived from *Pueraria lobata* on the osteoporotic rats. The data demonstrated the differentiation and mineralization of hBMSCs was significantly improved following the treatment of *Pueraria lobata*-derived exosome-like nanovesicles. This process was partly regulated through autophagy signaling (Zhan et al., 2023). Especially, to explore the role gut microbiota in the occurrence and development of osteoporosis. Zhang M. et al. (2025) isolated the EVs from L. salivarius. Their data demonstrated that *Lactobacillus salivarius* derived EVs could be transported to the site of osteoporotic lesions and alleviate the symptoms of osteoporosis (Zhang M. et al., 2025). And Shin et al. (2025) discovered that *Gardnerella vaginalis*-derived EVs increased the expressions of RANK, RANKL, and TNF-α in ovariectomy mice. This suggests *Gardnerella vaginalis*-derived EVs may be a risk factor for osteoporosis. Therefore, measures or methods to suppress them could help develop treatment methods for osteoporosis (Shin et al., 2025). The recent preclinical applications of EVs in osteoporosis, including the isolation method, the therapeutic effect, and the underlying mechanism, are summarized in Table 1. And the mechanisms of the therapeutic effect of EVs on osteoporosis were summarized in Figure 5.

**TABLE 1:** The recent progress on the therapeutic effect of EVs on osteoporosis.

Classification	Sources	Isolation method	Animal model	Therapeutic effect	Mechanism	References
EVs	ATDSCs	Multi-filtration System	Mouse	Inhibit osteoclast differentiation and reduce bone resorption	Regulating miR-21-5p	Lee et al. (2021)
EVs	Skeletal muscle	Total exosome Isolation reagent kit	Mouse	Improve osteogenic differentiation	Regulating glycolysis	Ma et al. (2023)
EVs	Gut microbiota	Ultracentrifugation	Mouse	Promote bone formation and inhibit bone resorption	Regulating gut microbiota	Liu et al. (2021)
EVs	*Escherichia coli*	Ultracentrifugation	Mouse	Promote osteogenic differentiation	Regulating BMP/SMAD	Liu et al. (2024a)
EVs	M2 macrophages	Ultracentrifugation	Mouse	Alleviate bone loss	Regulating JMJD3	Huang X. et al. (2024)
Exosomes	Yam	Ultracentrifugation	Mouse	Promote bone growth	Regulating BMP-2/p-p38	Hwang et al. (2023)
EVs	BMSCs	Ultracentrifugation	Mouse	Alleviate bone loss and promote bone formation	Regulating RAS/RAF1/MEK/ERK	Li F. et al. (2024)
EVs	Young osteocyte	Ultracentrifugation	Mouse	Promote bone mass and biomechanical strength	Regulating tropomyosin-1	Wang et al. (2024)
EVs	Skeletal muscles	Ultracentrifugation	Mouse	Regulate bone formation and bone resorption	—	Huang et al. (2023)
EVs	BMSCs	Ultracentrifugation	Mouse	Promote osteogenesis	—	Gui et al. (2024)
EVs	Human skeletal Muscle myoblasts	Differential centrifugation And ultracentrifugation	Mouse	Promote osteogenesis	Regulating miR-873–3p	Chen W. et al. (2024)
EVs	BMSCs	Differential centrifugation And ultracentrifugation	Mouse	Improve bone loss and cartilage damage	Regulating Wnt/β-catenin	Wang L. et al. (2023)
EVs	BMSCs	Differential centrifugation And ultracentrifugation	Mouse	Improve bone homeostasis	Regulating TGF-β/Smad 2/3-Wnt/β-catenin	Zhang Y. et al. (2024)
Exosomes	Umbilical cord	Ultracentrifugation	Rat	Promote the bone regeneration	—	Deluca et al. (2024)
EVs	Urine-derived Stem cells	Ultracentrifugation	Rat	Promote the osteoblast differentiation	Regulating HDAC4/HIF-1α/VEGFA	Zhang et al. (2023)
EVs	Defatted milk	Ultracentrifugation	Mouse	Regulate osteogenesis and osteoclastogenesis	—	Huang Q. et al. (2024)
EVs	MSCs	Ultracentrifugation	Mouse	Increase bone density	Regulating wnt/β-catenin	Wang Y. et al. (2022)
Exosomes	Oyster mantle	Ultracentrifugation	Rat	Promote osteogenic activity	Regulating PI3K/Akt/β-catenin	Hu et al. (2024)
Exosomes	Myocytes	Ultracentrifugation	Mouse	Promote osteogenesis	Regulating miR-92a-3p/PTEN/AKT	Xu et al. (2023)
Exosomes	Monocytic and MC3T3-E1 cell	Ultracentrifugation	Mouse	Inhibit osteoclasts	Regulating m6A methylation	Yang et al. (2023)
EVs	MSCs	Ultracentrifugation	Mouse	Regulate bone homeostasis	Regulating RANK-RANKL	Chang et al. (2024)
EVs	Bovine milk	Ultracentrifugation	Rat	Increase the bone mineral density	Regulating RUNX2	Go et al. (2021)
EVs	BMSCs	Ultracentrifugation	Rat	Promote osteoclast differentiation	Regulating miR-15b-5p	Xu et al. (2024)
EVs	BMSCs	Ultracentrifugation	Mouse	Promote osteogenic differentiation	Regulating wnt/β-catenin	Duan et al. (2023)
Exosomes	Cation-free kiwi fruit	Ultracentrifugation	Mouse	Promote osteogenesis	Regulating miR-92a-3p/PTEN/AKT	Xu et al. (2023)
Exosomes	Bone microvascular endothelial cells	Ultracentrifugation	Mouse	Inhibit osteoclasts	Regulating METTL14	Yang et al. (2023)
Exosomes	Recombinant probiotics *Escherichia coli* Nissle 1917	Ultracentrifugation	Mouse	Improve bone capacity	Regulating GSK3β	Kong et al. (2025)
EVs	Splenic pan T Cells	Sequential centrifugation	Mouse	Ameliorate bone loss	Regulating A2BR and PKA	Yang X. et al. (2025)
EVs	Mastocytosis	Density gradient fractionation	Mouse	Prevent osteoblastogenesis	Regulating RUNX2 and SMAD1/5	Kim et al. (2021)
EVs	Oral milk	Ultracentrifugation	Mouse	Ameliorate bone loss	Regulating gut microbiota	Hao et al. (2024)
EVs	BMSCs	Ultracentrifugation	Rat	Improve structure of alveolar bone	—	Sedik et al. (2023)
EVs	Sea buckthorn	Differential centrifugation	Mouse	Improve bone regeneration	Regulating miR168/LBH/RUNX2	Zhao Z. et al. (2025)
Extracellular Nanovesicles	Herba epimedium	Ultracentrifugation	Mouse	Repair mandibular defects	Regulating PI3k/Akt/mTOR	Han et al. (2025)
Exosomes	MSCs	Ultrafiltration	Mouse	Improve bone loss	Regulating CXCL9-CXCR3	Kang et al. (2024)
Exosomes	Skeletal muscle	Ultracentrifugation	Rat	Promote osteogenesis	—	Xing et al. (2024)
EVs	Morinda officinalis	Differential centrifugation And ultracentrifugation	Mouse	Promote osteoblast proliferation	Regulating MAPK	Cao et al. (2024)
Exosomes	Platelet lysate	Ultracentrifugation	Rat	Enhance bone anabolism	—	Zhang R. et al. (2024), Zheng et al. (2022)
EVs	Bovine milk	Ultracentrifugation	Mouse	Improve alveolar bone loss	Regulating RANKL/OPG	Silva et al. (2024)
Exosomes	Pueraria lobata	Ultracentrifugation	Rat	Improve bone regeneration	Regulating autophagy	Zhan, et al. (2023)
Exosomes	BMSCs	Ultracentrifugation	Mouse	Promote osteoblast proliferation	Regulating miR-150–3p	Silva, et al. (2024)
Exosomes	Osteoclasts	Ultracentrifugation	Rat	Inhibit bone formation	Regulating pyroptosis	Qiu, et al. (2021)
Exosomes	Macrophage	Ultracentrifugation	Mouse	Suppress osteoclast differentiation	—	Gao, et al. (2025)
EVs	Steleophaga plancyi	Differential velocity centrifugation	Rat	Promote osteogenic differentiation	—	Lu et al. (2025a)
EVs	*Lactobacillus* Salivarius	Ultracentrifugation	Rat	Regulate bone homeostasis	Regulating gut microbiota	Zhang W et al. (2025)
EVs	BMSCs	Ultracentrifugation	Mouse	Promote bone formation	Regulating miR-27a-3p/LMNB1	Geng et al. (2024)
Exosomes	Myoblasts	Ultracentrifugation	Mouse	Regulate bone metabolism	—	Tang J. S. et al. (2025)
EVs	Blood sample	Differential centrifugation	Rat	Alleviate bone loss	Regulating JNK/p38MAPK	Chen Y. et al. (2024)
Exosomes	Human umbilical vein endothelial cells	Ultracentrifugation	Mouse	Promote bone resorption	Regulating NF-κB	Mao et al. (2025)
Exosomes	Adipocyte	Differential centrifugation	Mouse	Improve bone microarchitecture	Regulating MAPK	Pi et al. (2025)
Exosomes	HUCMSCs	Ultracentrifugation	Mouse	Improve osteogenic differentiation	Regulating PI3K/AKT	Wang X. et al. (2023)
Exosomes	BMMSCs	Ultracentrifugation	Mouse	Improve osteogenesis	Regulating TAF15/RUNX2	Zhao M. et al. (2025)
Exosomes	Artesunate	Ultracentrifugation	—	Promote osteogenesis	Regulating TAF15-RUNX2	Huang et al. (2022)
Exosomes	Gardnerella vaginalis	Ultracentrifugation	Mouse	Decrease inflammation	Regulating RANK/RANKL	Shin et al. (2025)
EVs	BMMSCs	Ultracentrifugation	Mouse	Enhance osteoblast proliferation	Regulating PINK1/Parkin	Xu et al. (2024)
Exosomes	BMMSCs	Ultracentrifugation	Mouse	Suppress bone regeneration	Regulating miR-578/HMGA2	Li M. et al. (2024)
Exosomes	Serum of young rats	ExoQuick exosome precipitation kit	Rat	Improve the osteogenic differentiation	Regulating PTEN	Xun et al. (2021)

Abbreviations: AKT, protein kinase B; ATDSCs, adipose tissue-derived stem cells; A2BR, A2B receptor; BMP, bone morphogenetic protein; BMSCs, bone marrow stem cells; CXCR3, C-X-C motif chemokine receptor 3; ERK, extracellular signal-regulated kinase; EVs, extracellular vesicles; GSK3B, glycogen synthase kinase 3 beta; HDAC4, histone deacetylase 4; HIF1α, hypoxia-inducible factor 1α; HMGA2, high mobility group AT-hook 2; JMJD3, the Jumonji domain-containing protein-3; JNK, c-Jun N-terminal kinase; LMNB1, lamin B1; MAPK, mitogen-activated protein kinase; mTOR, mammalian target of rapamycin; MEK, Mitogen-activated protein kinase; METTL14, methyltransferase 14, N6-adenosine-methyltransferase non-catalytic subunit; RAS, rat sarcoma; RAF1, serine/threonine kinase; Wnt, Wingless/Integrated; TGF-β, transforming growth factor-β; VEGFA, vascular endothelial growth factor A; MSCs, mesenchymal stem cells; PI3K, phosphoinositide-3-kinase; NF-κB, nuclear factor kappa-B; OPG, osteoprotegerin; PTEN, phosphatase and tensin homolog; PKA, protein kinase A; PINK1, PTEN, induced putative kinase 1; RANK, receptor activator of nuclear factor-kappa B; RUNX2, RUNT, family transcription factor 2; RANKL, receptor activator ofnuclear factor kappa-B ligand; SMAD, SMAD, Family Member 1.

## 6 The diagnostic applications of EVs in osteoporosis

It has been demonstrated that EVs have the capacity to isolate from diverse body fluids, including urine, blood, saliva, and ascites (Wang et al., 2025; Pan et al., 2024; Peng et al., 2025; Gorini et al., 2025). Furthermore, EVs have been shown to encapsulate a range of various active molecules, including DNA, RNA, and proteins. These can be identified as the biomarkers or therapeutic targets for the diseases, including osteoporosis (Abebaw et al., 2024; Luo et al., 2025). It has been demonstrated that RNAs are involved in the regulation of the functions of osteoblasts and osteoclasts. Because of the characteristic of RNAs, including its relative stability in bodily fluids and its ease of detection through various RNA detection techniques, including high-throughput sequencing and RT-PCR. EVs derived RNAs have rapidly developed into a potential diagnostic biomarker for osteoporosis. For example, Shi et al. (2022) isolated and analyzed the exosome samples from postmenopausal women, thereby discovering that microRNAs (miRNAs) 324-3p, 766-3p and 1247-5p exhibited a strong correlation with bone mineral density (BMD). Furthermore, microRNA 330-5p was found to have a promoting effect on alkaline phosphatase (ALP) activity in human bone marrow stromal cells (hBMSCs). These findings suggest that these specific miRNAs may serve as valuable diagnostic biomarkers for postmenopausal osteoporosis (Shi et al., 2022).

Similarly, Gao et al. (2024) collected and analyzed the plasma-derived exosomes of 272 female volunteers with the objective of identifying the early biomarkers of postmenopausal osteoporosis. The data demonstrated the presence of 70 differentially expressed exo-miRNAs, and further lasso-logistic regression analysis indicated that hsa-let-7d-3p, hsa-miR-24-3p and hsa-miR-550a-3-5p exhibited statistical correlation with postmenopausal osteoporosis, suggesting their potential as biomarkers for this condition (Gao et al., 2024). Furthermore, Shao et al. (2020) conducted a comparative analysis of exosomal miRNA profiles between 12 menopausal osteoporosis patients and six menopausal individuals utilizing high-throughput sequencing of microRNAs. It was established that the differentially expressed RNAs between two groups were associated with the progression of osteoporosis (Shao et al., 2020). In the study by Pepe et al. (2022) a comparison was made of the number and the active molecules of plasma EVs between osteoporosis patients and healthy controls. The data demonstrated that the number of RANKL^+^ EVs in osteoporosis patients were higher than that in healthy controls, and the expression of miR-1246 was significantly increased in EVs from patients with osteoporosis, indicated that RANKL^+^ EVs and exosomal miR-1246 may be the potential biomarkers for osteoporosis (Pepe et al., 2022). Sun et al. (2023) analyzed the exosomal miRNAs profiles of 12 PMOP patients and 12 healthy controls by the next-generation sequencing and bioinformatics analysis. The results demonstrated that there were 27 differentially expressed miRNAs between the two groups. The combination of microRNAs (miR-34a-5p + miR-9-5p + miR-98-5p) was identified as a potential biomarker for the diagnosis of postmenopausal osteoporosis (Sun et al., 2023). Renuka et al. (2025) identified exosomal miR-19b as a diagnostic biomarker for estrogen-related osteoporosis, found that this type of exosomal miR-19b could regulate the level of IGF-1 and participate in the progression of osteoporosis (Renuka et al., 2025).

In addition to miRNAs, various forms of circular RNAs and long non-coding RNAs (lncRNAs) have been also identified as the diagnostic biomarkers for osteoporosis. For example, Zhi et al. (2021) identified a type of circular RNA (circRNA), termed Hsa_circ_0006859, which exhibited significantly higher levels of expression in patients suffering from osteoporosis in comparison to healthy control subjects. This particular circRNA has been found to possess the capacity to impede osteogenesis and to promote adipogenesis, thus serving as an indicative biomarker for postmenopausal osteoporosis. This process was demonstrated to regulate by sponging miR-431-5p, and further upregulate the expression of ROCK1 (Zhi et al., 2021). Fu et al. (2022) conducted an analysis of the circRNA profiles of BMSC-derived exosome samples obtained from patients diagnosed with postmenopausal osteoporosis. Their findings revealed the presence of 237 upregulated circRNAs and 279 downregulated circRNAs in patients with postmenopausal osteoporosis compared with control group. The results of this study suggest that circRNAs from EVs may hold significant potential as a biomarker for the diagnosis or as a therapeutic target for patients with postmenopausal osteoporosis. The further mechanisms were demonstrated to involved the regulation of autophagy, PI3K-Akt signaling, FoxO signaling, and MAPK signaling (Fu et al., 2022). Han et al. (2020) identified Hsa_circ_0006859 from serum samples of patients by circRNA microarray and qRT-PCR analysis. It was determined that Hsa_circ_0006859 demonstrated a high degree of specificity between osteoporosis patients and healthy individuals, and participated in the processes of osteogenesis and adipogenesis by regulating ROCK1 (Han et al., 2020). Furthermore, Wang J. et al. (2022) investigate the diagnostic efficacy of long non-coding RNA (lncRNA) in osteoporosis. The data demonstrated that there were 286 differentially expressed lncRNAs between postmenopausal osteoporosis patients and healthy controls. Further RT-qPCR and microarray identified five lncRNAs that exhibited a strong correlation with the progression of osteoporosis, which can regulate the several key signaling pathways, including the MAPK and PI3K-Akt pathways (Wang Y. et al., 2022). In order to comprehend the diagnostic efficacy of transfer RNA-derived fragments (tRFs) in osteoporosis, Zhang et al. (2018) analyzed the exosomes from 40 healthy people and 40 osteoporosis patients using small RNA sequencing and qPCR analysis. The analysis of the data yielded the discovery of 29 differentially expressed tRFs. Of these, tRF-25, tRF-38 and tRF-18 exhibited a high degree of accuracy in the diagnosis of osteoporosis, thus suggesting their potential as biomarkers for this condition (Zhang et al., 2018).

Cross-cell communication of EVs carrying non-coding RNAs (ncRNAs) has opened up a new dimension for the precise diagnosis of osteoporosis (Huang W. et al., 2026). It is important to note that microRNAs, circular RNAs (circRNAs) and long non-coding RNAs (lncRNAs) form a complex regulatory axis involving a “lncRNA/circRNA-miRNA” cascade (Yuan et al., 2026). Different bone-related cells, including osteoblasts, osteoclasts and BMSCs, secrete EVs that carry specific ncRNA ‘cargo’ and reflect the pathological state of cells from different sources (Long et al., 2026). Therefore, detecting EV-derived ncRNAs in plasma or serum has become a feasible strategy for the non-invasive monitoring of bone metabolism dynamics (Li Q et al., 2026). Additionally, the study revealed discrepancies in the expression profiles of non-coding RNA (ncRNA) in different bone regions. This suggests that the development of specific combinations of non-coding RNA markers has the potential to identify sites with a high risk of fracture (Chakraborty and Banerjee, 2025).

**FIGURE 6:**
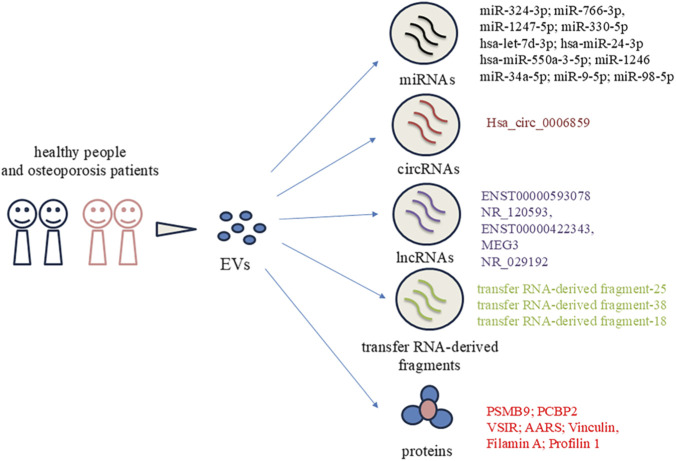
The summary of the diagnostic applications of EVs in osteoporosis.

In order to identify potential therapeutic protein targets for osteoporosis, Chen et al. (2020) analyzed the protein profiles of exosome samples from 60 participants using quantitative proteomics technology. This analysis identified four proteins, namely, PSMB9, PCBP2, VSIR and AARS, which were found to be associated with osteoporosis (Chen et al., 2020). Huo et al. (2019) isolated the microvesicles from the serum samples in osteoporosis, osteopenia and normal group. The results of the comparative proteomics analysis demonstrated that the presence of 19 proteins that were found to be upregulated, as well as five proteins that were downregulated. And Vinculin, Filamin A, and Profilin one were identified to potential biomarkers for the osteoporosis (Huo et al., 2019) (Figure 6).

## 7 Conclusion and prospects

It is evident that EVs possess a number of advantageous characteristics, including their convenient source, low immunogenicity and multiple biological functions such as anti-inflammatory, immune regulatory and tissue repair properties. In addition, EVs can be modified through a variety of physical and chemical methods, thereby demonstrating their significant potential in the treatment of osteoporosis. Currently, there are still several application limitations that need to be considered. (1) Classification and heterogeneity of EVs. EVs have been found to be capable of being isolated from a variety of cell types, including mesenchymal stem cells (MSCs) and tumor cells, as well as different bodily fluids, such as blood, saliva and urine. At the same time, EVs have been shown to contain DNAs, RNAs, and proteins. The heterogeneity of EV sources and the diversity of their compositions give rise to quality issues that must be addressed. In addition, currently the classification of EVs is based on two key factors: their sources and their diameters. This rudimentary classification method is unable to differentiate between the characteristics and functionality of EVs. The International Society of EVs (ISEV)’s MISEV Guidelines has stated that a more accurate approach would be to describe EVs based on their “physical properties” and “biochemical characteristics”. This guideline is unable to address the issue of standardized methods for large-scale isolation, purification, characterization, and storage of EVs. The prevailing isolation methodologies for EVs primarily encompass ultracentrifugation and ultrafiltration, which complicates the assurance of EV purity. These elements impact the application of EVs in the context of osteoporosis. The establishment of relevant legislative standards, including the sources, isolation methods, and identification methods of EVs subtypes, may provide a solution to this problem. Exploration of new sources of EVs, for example, attempting to obtain EVs in large quantities from milk, plants (such as yam, oysters). Preliminary studies have demonstrated that these sources of EVs have the potential to promote bone formation. Similarly, it may be advisable to stimulate target cells in low oxygen or inflammatory environments to enhance the therapeutic effect of EV secretion. (2) Osteoporosis is a long-term and multifactorial disease, and current animal modeling methods are not well suited to reflecting the pathological state of human osteoporosis. Concurrently, the present non-clinical study administration regimens of EVs for osteoporosis demonstrate significant variation, and clinical trials are infrequent, hindering the further verification of their effectiveness and safety. It is challenging to provide a comprehensive evaluation of the safety and efficacy of EVs in the treatment of osteoporosis. It is recommended that multiple dosing regimens be evaluated in conjunction, and that large-scale clinical trials be conducted at multiple centers. This approach is recommended on the basis of mounting non-clinical research on the application of EVs in osteoporosis, with the objective of fully verifying the safety and efficacy of EVs in treating osteoporosis and expediting the application of EVs in this field. (3) There are differences in the absorption efficiency of EVs among different organs or cells. EVs possess a specific targeting capability that influences their distribution within the body. Different administration routes, the size and surface characteristics of EVs also affect their distribution in the body and cellular absorption. In order to improve the targeting and therapeutic efficacy of EVs in the treatment of osteoporosis, it is necessary to modify the surface of EVs with bone-specific receptors, load bone-specific proteins or RNA, and achieve precise drug delivery. Alternatively, using nanomanufacturing using nano processing technology to load EVs into biological materials, including hydrogels, 3D printing scaffolds or electrospun nanofibers, can ensure the bioactivity of EVs is protected and the slow and controlled release of EVs is realized. (4) Further research is required to ascertain the long-term biosafety of EVs. At present, research is mostly focused on short-term animal experiments, and the long-term distribution, metabolism, and potential risk data of EVs in the body are not yet clear. In order to promote the clinical research and application of EVs in osteoporosis, it is necessary to establish quality control standards and a long-term safety evaluation system for osteoporosis drugs targeting EVs.
